# Anti-Inflammatory and Reactive Oxygen Species Suppression through Aspirin Pretreatment to Treat Hyperoxia-Induced Acute Lung Injury in NF-κB–Luciferase Inducible Transgenic Mice

**DOI:** 10.3390/antiox9050429

**Published:** 2020-05-15

**Authors:** Chuan-Mu Chen, Yu-Tang Tung, Chi-Hsuan Wei, Po-Ying Lee, Wei Chen

**Affiliations:** 1 Department of Life Sciences, and Ph.D. Program in Translational Medicine, National Chung Hsing University, Taichung 402, Taiwan; chchen1@dragon.nchu.edu.tw (C.-M.C.); crystalwei823@gmail.com (C.-H.W.); 2 The iEGG and Animal Biotechnology Center, and the Rong Hsing Research Center for Translational Medicine, National Chung Hsing University, Taichung 402, Taiwan; 3 Graduate Institute of Metabolism and Obesity Sciences, Taipei Medical University, Taipei 110, Taiwan; f91625059@tmu.edu.tw; 4 Nutrition Research Center, Taipei Medical University Hospital, Taipei 110, Taiwan; 5 Cell Physiology and Molecular Image Research Center, Wan Fang Hospital, Taipei Medical University, Taipei 110, Taiwan; 6 Division of Plastic Surgery, Department of Surgery, Cathay General Hospital, Taipei 280, Taiwan; poying1018@gmail.com; 7 Division of Pulmonary and Critical Care Medicine, Chia-Yi Christian Hospital, Chiayi 600, Taiwan

**Keywords:** aspirin, hyperoxia, acute lung injury, NF-κB–luciferase inducible transgenic mice, in vivo imaging system (IVIS), anti-inflammation

## Abstract

Acute lung injury (ALI), a common cause of morbidity and mortality in intensive care units, results from either direct intra-alveolar injury or indirect injury following systemic inflammation and oxidative stress. Adequate tissue oxygenation often requires additional supplemental oxygen. However, hyperoxia causes lung injury and pathological changes. Notably, preclinical data suggest that aspirin modulates numerous platelet-mediated processes involved in ALI development and resolution. Our previous study suggested that prehospital aspirin use reduced the risk of ALI in critically ill patients. This research uses an in vivo imaging system (IVIS) to investigate the mechanisms of aspirin’s anti-inflammatory and antioxidant effects on hyperoxia-induced ALI in nuclear factor κB (NF-κB)–luciferase transgenic mice. To define mechanisms through which NF-κB causes disease, we developed transgenic mice that express luciferase under the control of NF-κB, enabling real-time in vivo imaging of NF-κB activity in intact animals. An NF-κB-dependent bioluminescent signal was used in transgenic mice carrying the luciferase genes to monitor the anti-inflammatory effects of aspirin. These results demonstrated that pretreatment with aspirin reduced luciferase expression, indicating that aspirin reduces NF-κB activation. In addition, aspirin reduced reactive oxygen species expression, the number of macrophages, neutrophil infiltration and lung edema compared with treatment with only hyperoxia treatment. In addition, we demonstrated that pretreatment with aspirin significantly reduced the protein levels of phosphorylated protein kinase B, NF-κB and tumor necrosis factor α in NF-κB–luciferase^+/+^ transgenic mice. Thus, the effects of aspirin on the anti-inflammatory response and reactive oxygen species suppressive are hypothesized to occur through the NF-κB signaling pathway. This study demonstrated that aspirin exerts a protective effect for hyperoxia-induced lung injury and thus is currently the drug conventionally used for hyperoxia-induced lung injury.

## 1. Introduction

Acute respiratory distress syndrome (ARDS), or acute lung injury (ALI), is a common and devastating syndrome contributing to serious morbidities and mortality in critically ill patients. The pathophysiologic features of ARDS include dysregulated inflammation, inappropriate accumulation and activity of leukocytes and platelets, uncontrolled activation of coagulation pathways, and altered permeability of the alveolar endothelial and epithelial barriers [1,2,3], leading to the impairment of oxygenation and subsequent respiratory failure. Therefore, patients with ARDS may require mechanical ventilation support with a high concentration of inspired oxygen [4]. However, prolonged hyperoxic exposure can exacerbate the pathogenic processes within the lung [5], cause the generation of reactive oxygen species (ROS) [6] and increase apoptotic signaling [7], all of which may result in hyperoxia-induced ALI [8,9], causing pathological changes resembling ARDS in animal models [10]. Although numerous promising therapies have effectively prevented ARDS in experimental models, successful translation to clinical applications is still lacking [11,12,13].

Platelets play a major role in the inflammatory response leading to ARDS. The possible mechanisms by which platelets contribute to ARDS include the activation of endothelial cells by release of proinflammatory mediators [14,15,16] and the adherence of platelets to lung capillary endothelial cells, thereby activating attached leukocytes [17]. Aspirin, an antiplatelet agent, is commonly used in clinical practice. Experimental studies have shown that in models of transfusion-related ALI, lipopolysaccharide-induced ALI and hydrochloric acid-induced ALI, aspirin can prevent or treat ARDS by reducing neutrophil activation and recruitment to the lung, tumor necrosis factor α (TNF-α) expression in pulmonary intravascular macrophages, plasma thromboxane B2 levels and platelet sequestration [18,19,20,21,22,23]. However, experimental studies on the use of aspirin for prevention or treatment of hyperoxia-induced ALI are limited [24]. Cox et al. [25] showed that aspirin-triggered resolvin D1 treatment significantly reduced lung edema, permeability, inflammation and apoptosis in a murine model of prolonged hyperoxic exposure. Moreover, our group reported in a study of 1149 patients from the validating acute lung injury markers for diagnosis cohort, that prehospital aspirin use was independently associated with a decreased risk of ARDS even after adjustment for the propensity of prehospital aspirin use [26]. Based on these findings, further investigation of the mechanisms of the protective effects of aspirin against hyperoxia-induced ALI is crucial.

Using an in vivo imaging system (IVIS), we investigated the mechanisms of aspirin’s anti-inflammatory and antioxidant effect on hyperoxia-induced ALI in nuclear factor κB (NF-κB)–luciferase transgenic mice. We hypothesized that low or high doses of aspirin prevent hyperoxia-induced lung injury through decreased oxidative stress and inflammation.

## 2. Materials and Methods

### 2.1. Animals

The transgenic mice (aged 8 weeks) were given a standard laboratory diet and distilled water ad libitum and were maintained on a 12-h light–dark cycle at 24 ± 2 °C. This study was conducted according to institutional guidelines and approved by the Institutional Animal Care and Utilization Committee of National Chung Hsing University, Taichung, Taiwan, and the study conformed to the guidelines of the protocol IACUC Approval No. 102-77 approved by the IACUC ethics committee. Part I: FVB (friend leukemia virus B) mice were randomly assigned to four groups for treatment (*n* = 6): (1) normal control, (2) treatment with PBS (phosphate buffered saline) followed by exposure to 24 h of hyperoxia (24-h group), (3) treatment with PBS followed by exposure to 48 h of hyperoxia (48-h group) and (4) treatment with PBS followed by exposure to 72 h of hyperoxia (72-h group). Part II: The homozygous transgenic mice (NF-κB–luciferase^+/+^) were randomly assigned to four groups for pretreatment (*n* = 6): (1) negative control, (2) pretreatment with PBS for 3 days followed by exposure to 72 h of hyperoxia (mock group), (3) pretreatment with a low dose of aspirin (12.5 μg/g) for 3 days followed by exposure to 72 h of hyperoxia (aspirin-L group) and (4) pretreatment with a high dose of aspirin (100 μg/g) for 3 days followed by exposure to 72 h of hyperoxia (aspirin-H group). At the end of the pretreatment, mice were exposed to hyperoxia conditions for 72 h. At the end of the experiment, each mouse was anesthetized, and pulmonary tissues were collected for bronchoalveolar lavage, pathological histology and protein extraction according to previously established protocols [27].

### 2.2. Murine Model of Hyperoxia-Induced Acute Lung Injury

The hyperoxia-exposed mice were housed in humidified 99% oxygen in a hyperoxia chamber (36 cm × 20 cm × 20 cm) with normobaric pressure. The oxygen levels were stable (97%–99%) and monitored every hour during the housing light cycle with an oxygen analyzer (MiniOX I, MSA Canada, Inc., Etobicoke, ON, Canada) [28]. The mice were sacrificed after exposure to oxygen.

### 2.3. Imaging of Luciferase Activity

Imaging was performed using the IVIS Imaging System 200 Series (Xenogen Corp., Alameda, CA, USA) with the camera set at the highest sensitivity. NF-κB-luciferase^+/+^ transgenic mice were injected intraperitoneally with 150 mg/kg of luciferin and anesthetized with isoflurane. After 5 min, the mice were placed supine in the chamber and imaged for 1–3 min with the IVIS Imaging System. Photons were quantified using Living Image^®^ software (Xenogen Corporation), and the signal intensity was expressed as photons/s/cm^2^.

### 2.4. Reverse Transcription Polymerase Chain Reaction

The total RNA of the pulmonary tissue was extracted using TRIzol reagent (Invitrogen) following the protocol specified by the manufacturer. Total RNA (2 μg) was resuspended in 9 μL of diethylpyrocarbonate-treated water, and the first strand of cDNA was synthesized with a total volume of 20 μL of random primers and ImProm-IITM reverse transcriptase. The reaction was performed at 42 °C for 1 h. For further PCR amplification, an aliquot (1:10) of the RT product was adjusted to produce 0.1 μg of each primer, and additional buffer was added to yield a final volume of 20 μL. Aliquots of the reverse transcriptase mix were used for PCR amplification of luciferase and β-actin. The amplified RT–PCR products were subjected to electrophoresis in a 1.5% agarose gel for 22 min. The cDNA of β-actin was used as an internal control.

### 2.5. Western Blot Analysis

Expression of pulmonary tissue protein was measured using western blot analysis. Pulmonary tissues were homogenized in 270 μL of radioimmunoprecipitation assay buffer (5-mM Tris–HCl [pH 7.4], 0.15-M NaCl, 1% NP40, 0.25% sodium deoxycholate, 5 mM ethylenediaminetetraacetic acid and 1-mM ethylene glycol-bis [2-aminoethyl-ether]-*N*, *N*, *N*, *N*-tetraacetic acid) with 30 μL of phosphatase inhibitor. The homogenates were centrifuged at 13,000 rpm for 30 min at 4 °C. Protein (50 μg) was then separated using sodium dodecyl sulfate–polyacrylamide gel electrophoresis in a 15% polyacrylamide gel and electrotransferred onto a polyvinylidene difluoride membrane. The membranes were incubated in blocking solution (5% bovine serum albumin) at room temperature for 1 h. The membranes were then incubated with primary antibody (neuropilin 1 [NRP-1], Clara cell 10 kilodalton protein [CC10], NF-κB, interleukin 8 [IL-8], interleukin 1β, TNF-α, chemokine ligand 5 and β-actin) at room temperature for 2 h. After washing, the membranes were incubated with either a goat antirabbit or goat antimouse immunoglobulin peroxidase–conjugated secondary antibody directed against the primary antibody at room temperature for 1 h. The membranes were developed using an enhanced chemiluminescence detection system as described elsewhere [29].

### 2.6. Pathological Histology

Lung tissues were fixed in 4% formaldehyde overnight, embedded in paraffin, and cut into sections for hematoxylin and eosin (H&E) staining as described elsewhere [30].

### 2.7. Measurement of ROS Generation

Generation of ROS in the perfused lungs was monitored with 2′,7′-dichlorodihydrofluorescein diacetate (H2DCF-DA) fluorescence as described elsewhere [31]. After internalization, the acetate group from the nonfluorescent molecule is cleaved using intracellular esterases to form 2′,7′-dichlorodihydrofluorescein, which serves as a substrate for intracellular ROS to generate highly fluorescent dichlorofluorescein. Fluorescence was measured with a spectrofluorometer at 480-nm excitation and 530-nm emissions. Data are expressed as relative fluorescence units for each cell.

### 2.8. Analysis of Airway Inflammation in Bronchoalveolar Lavage Fluid

Bronchoalveolar lavage fluid (BALF) was collected using 500 μL of sterile endotoxin-free saline to wash the lungs. BALF cells were collected with centrifugation at 500× *g* at 4 °C. The number of BALF cells was determined using a hemocytometer [28].

### 2.9. Statistical Analysis

Experimental values are expressed as the mean ± standard error. Differences in experimental groups were determined using the Student’s *t*-test or one-way analysis of variance followed by the post hoc Duncan’s test. Differences with *p* < 0.05 were considered statistically significant.

## 3. Results

### 3.1. Time Course of Lung Damage Induced by Hyperoxia in Mouse Lung in NF-κB-Luciferase^+/+^ Transgenic Mice

**Figure 1:**
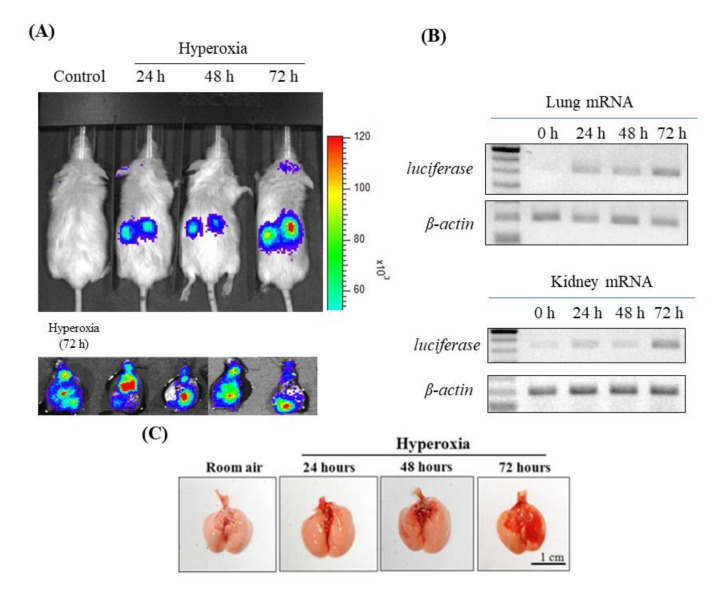
(**A**) Bioluminescence imaging of kidney and lung tissues; (**B**) luciferase mRNA expression of lung and kidney tissues; and (**C**) histopathological analyses of lung tissues in NF-κB–luciferase transgenic mice after induction of hyperoxia for 0, 24, 48 and 72 h.

This study investigated the time courses of inflammation and tissue damage after hyperoxia in FVB mouse lungs and kidneys (Figure 1A). To achieve this goal, we used hyperoxia exposure periods of 24, 48 and 72 h. After 72 h of hyperoxia, the signal and the mRNA expression of luciferase from the lung and kidney tissues were more obvious in the 72-h group than in the 24-h, 48-h and normal control groups (Figure 1A,B). In addition, lung erythematous swelling was more obvious in the 72-h group than in the 24-h, 48-h and normal control groups (Figure 1C).

### 3.2. Time Course of Hyperoxia in Relation to Alveolar Injury and Inflammation in NF-κB-Luciferase^+/+^ Transgenic Mice

**Figure 2:**
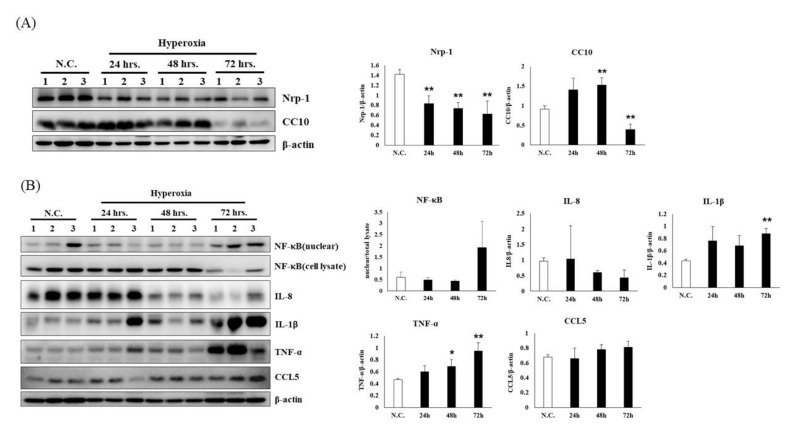
Changes in the protein expression levels for (**A**) alveolar injury and (**B**) inflammation of lung tissues in NF-κB–luciferase transgenic mice after induction of hyperoxia for 0, 24, 48 and 72 h. The reported values are expressed as the mean ± SEM (*n* = 6). Values assigned to letters (*, **) are significantly different at *p* < 0.05 and are determined using Duncan’s multiple range test.

Figure 2A shows NRP-1 and CC10 in the lung tissue of mice exposed to normoxia (normal control group) or hyperoxia (24, 48 and 72 h). NRP1 is a transmembrane glycoprotein that acts as a coreceptor for numerous extracellular ligands including class III/IV semaphorins, certain isoforms of vascular endothelial growth factor and transforming growth factor beta. The NRP-1 expression in the 24-h, 48-h and 72-h groups decreased by 67%, 79% and 77%, respectively, compared with that in the normal control group. CC10 is the major product of nonciliated bronchiolar epithelial cells (Clara cells) and exhibits anti-inflammatory properties including inhibition of phospholipase A2 and phospholipase C. The CC10 in the 72-h group decreased by approximately 87% compared with the normal control group, but no significant differences were observed among the 24-h and 48-h groups. Thus, the CC10 expression decreased after 72 h of hyperoxia.

TNF-α increased by 29% after 24 h, by 48% after 48 h and by 103% after 72 h of hyperoxia compared with the normal control group (Figure 2B). In addition, Figure 2B indicates an obvious increase in IL-1β expression from 72 h compared with the normal control group. The results show that TNF-α progressively increased after 24 to 72 h of hyperoxia. TNF-α is one of the cytokines involved in systemic inflammation and is one of the cytokines that plays a role in the acute phase reaction. It is produced chiefly by activated macrophages, although it can be produced by other cell types such as CD4+ lymphocytes, NK cells, neutrophils, mast cells, eosinophils and neurons. Figure 2B reveals a slight decrease in IL-8 expression from 48 to 72 h compared with the normal control group. This finding is unexpected because most published data indicate that at high levels of hyperoxia, rodents exhibit high mortality by 72–96 h [32]. The burden of IL-8 was progressive between 0 and 24 h of hyperoxia exposure. Endothelial cells store IL-8 in their storage vesicles. When endothelial cells are seriously damaged, IL-8 is released. Thus, hyperoxia caused damage to endothelial cells from 48 to 72 h. Notably, after 72 h of hyperoxia, histological evidence of lung damage was observed. Therefore, we suggest that lung tissue damage induced by hyperoxia occurred mainly at 72 h.

### 3.3. Inhibition of Hyperoxia-Induced NF-κB Activation by Using Aspirin Pretreatment in NF-κB–Luciferase^+/+^ Transgenic Mice

**Figure 3:**
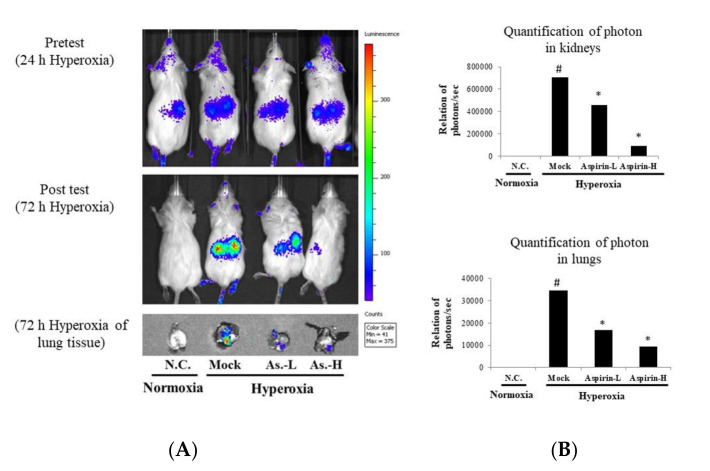
(**A**) Bioluminescence imaging of kidney and lung tissues and (**B**) photon quantification of kidney and lung tissues in NF-κB–luciferase transgenic mice after induction of hyperoxia for 72 h in the normal control (NC), mock, aspirin-L and aspirin-H groups. The reported values are expressed as the mean ± SEM (*n* = 6). ^#^ *p* < 0.05 vs. NC group. * *p* < 0.05 vs. mock group.

NF-κB–luciferase^+/+^ transgenic mice carry a luciferase gene driven by the NF-κB promoter. Therefore, luciferase activity reflects NF-κB activity [33,34]. After 72 h of hyperoxia exposure, the luciferase signal from the lung tissue was quantified using the IVIS software (Figure 3). Hyperoxia stimulated the luminescent signal in the lung tissue, but the luciferase signals in the aspirin-L group and aspirin-H group were significantly decreased compared with those in the mock group in both the lungs and kidneys. Thus, pretreatment with either 12.5 or 100 μg/g body weight of aspirin for 3 days in NF-κB–luciferase^+/+^ transgenic mice significantly protected against acute lung injury and systemic oxidative stress caused by hyperoxia (FiO_2_ > 95%).

### 3.4. Effect of Pretreatment on Histological Changes in the Lung in NF-κB–Luciferase^+/+^ Transgenic Mice

**Figure 4:**
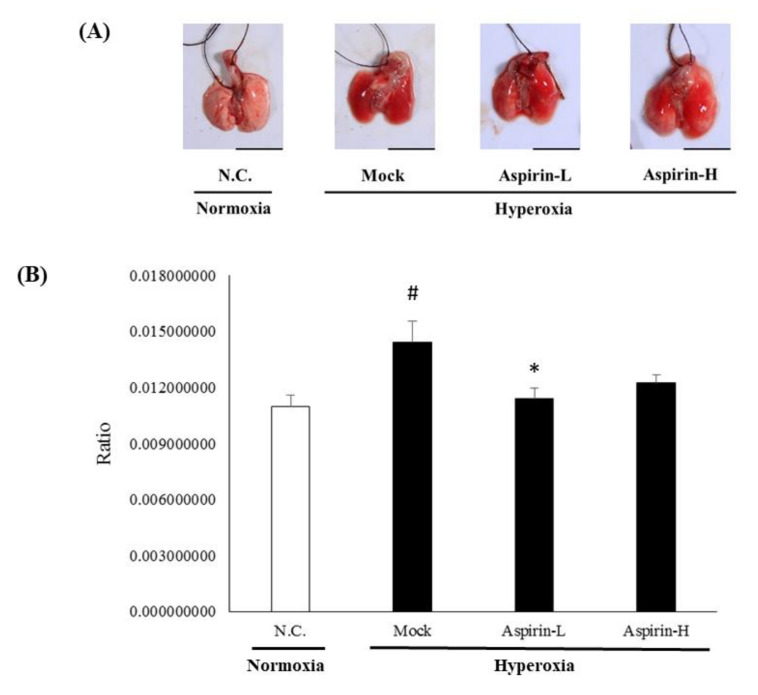
(**A**) Gross appearance of lung and (**B**) lung wet-to-dry weight ratio analysis in NF-κB–luciferase transgenic mice after the induction of hyperoxia for 72 h in the normal control (NC), mock, aspirin-L and aspirin-H groups. The reported values are expressed as the mean ± SEM (*n* = 6). ^#^ *p* < 0.05 vs. NC group. * *p* < 0.05 vs. mock group.

**Figure 5:**
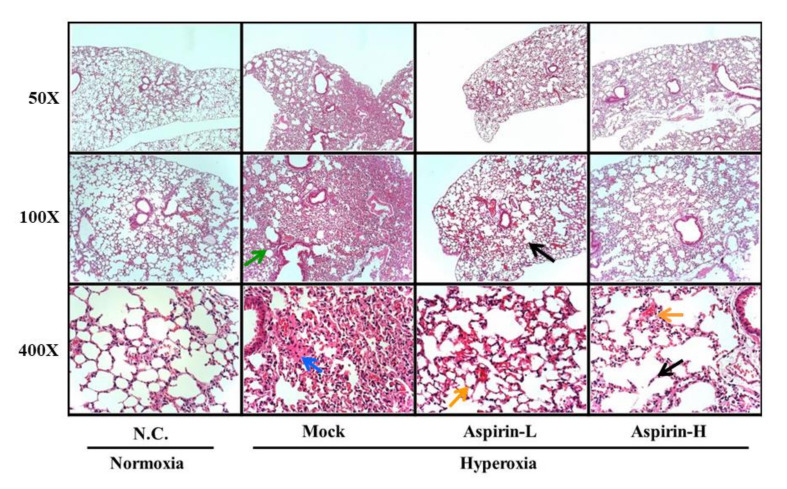
Histopathological analyses of lung tissues in the normal control (NC), mock, aspirin-L and aspirin-H groups. Low magnification (50×) images of lung tissues are shown in the upper panel. Medium magnification (100×) images of lung tissues are shown in the upper panel. High magnification (400×) images are shown in the lower panel. Green: neutrophil aggregation; blue: pulmonary edema; orange: erythrocyte aggregation; black: alveolar collapse.

Lung erythematous swelling was more obvious in the mock and aspirin-H groups (Figure 4A). As shown in Figure 4B, mice in the aspirin-L group exhibited significantly lower lung indices (lung weight/body weight) than those in the mock group. Histopathological examination of the lungs was performed after 72 h of hyperoxia. Pulmonary edema and alveolar infiltration of neutrophils were evident in the mock group (Figure 5). However, pretreatment with either a low or high dose of aspirin reduced neutrophil infiltration and lung edema.

### 3.5. Effects of Aspirin on the Generation of ROS and the Number of Macrophages in NF-κB–Luciferase^+/+^ Transgenic Mice

**Figure 6:**
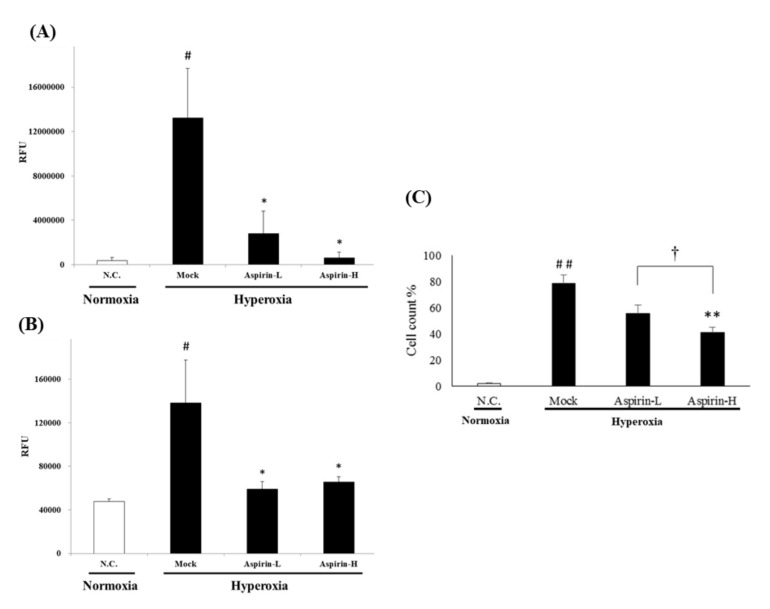
Quantification of reactive oxygen species (ROS) production in extracellular fluid (**A**) and intracellular fluid (**B**); the number of macrophages (**C**) of the bronchoalveolar lavage fluid (BALF) and from NF-κB–luciferase transgenic mice after induction of hyperoxia for 72 h in the normal control (NC), mock, aspirin-L and aspirin-H groups. The reported values are expressed as the mean ± SEM (*n* = 6). ^#^ *p* < 0.05, ^##^ *p* < 0.01 vs. NC group. * *p* < 0.05, ** *p* < 0.01 vs. mock group. † *p* < 0.05 vs. aspirin-L group.

The generation of ROS in the BALF was analyzed using H2DCF-DA fluorescence. A significant increase in the generation of extracellular and intracellular ROS in the BALF was observed in the mock group compared with the negative control group (Figure 6A,B). However, pretreatment with either a low dose or a high dose of aspirin significantly reduced the generation of ROS compared with the mock group. Macrophages are prominent cellular effectors of innate immune defense, and the numbers of macrophages in the BALF were analyzed using Liu’s stain. A significant increase in the numbers of macrophages (cell counts%) in the BALF was observed in the mock group compared with the negative control group (Figure 6C). However, pretreatment with either a low or high dose of aspirin significantly reduced the numbers of macrophages (cell counts%) compared with the mock group.

### 3.6. Effects of Aspirin on the Inflammatory Signaling in NF-κB-Luciferase^+/+^ Transgenic Mice

**Figure 7:**
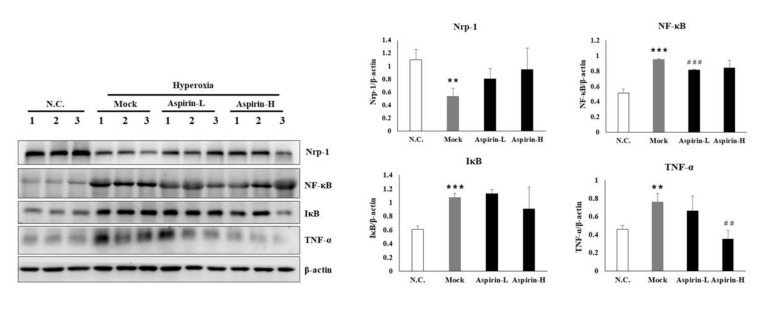
Protein expression of inflammatory signaling in NF-κB–luciferase transgenic mice after induction of hyperoxia for 72 h in the normal control (NC), mock, aspirin-L and aspirin-H groups. The reported values are expressed as the mean ± SEM (*n* = 6). ^##^ *p* < 0.01, ^###^ *p* < 0.001 vs. NC group. ** *p* < 0.01, *** *p* < 0.001 vs. mock group.

Due to ROS, hyperoxia can exacerbate organ failure through cellular injury. Therefore, a better understanding of the signal transduction pathways in hyperoxia may provide the basis for effective therapeutic interventions. The major biologic effects of hyperoxia include cell death, stress responses, inflammation and modulation of cell growth. NF-κB is among the major signaling pathways ostensibly involved, which converge, ultimately, to the expression of various stress response genes, cytokines and growth factors. In this study, inflammation was analyzed (Figure 7). The protein expression NRP-1 was markedly decreased in the mock group compared with the normal control group. However, pretreatment with a low dose of aspirin slightly increased the protein expression level of NRP-1. A significant increase of NF-κB was observed in the mock group compared with the normal control group. However, pretreatment with either a low or high dose of aspirin significantly reduced the protein expression of NF-κB compared with the mock group. The protein expression level of TNF-α was markedly increased in the mock group compared with the normal control group. Pretreatment with a high dose of aspirin significantly reduced the protein level of TNF-α. Thus, pretreatment with either low or high doses of aspirin could reduce the transcription of proinflammatory genes, limiting inflammation and regulating the extent of lung injury.

## 4. Discussion

The NF-κB–luciferase^+/+^ transgenic mice carry the luciferase gene driven by the NF-κB promoter. This provides a useful animal model for noninvasive detection of the inflammatory levels in a living organism. In this study, we induced lung damage in NF-κB–luciferase^+/+^ transgenic mice using hyperoxia conditions (FiO_2_ > 95%) to establish a model to evaluate aspirin’s protective effects against lung injury. Due to either protective effects, the luciferase signals of aspirin were significantly decreased compared with the mock group. In this study, we investigated the time courses of inflammation and lung damage after hyperoxia in the mouse lung. Groups of FVB mice were exposed to >95% oxygen in a chamber for 24, 48 or 72 h. The controls were subjected to normoxia. IL-8 was observed to increase after 24 h of hyperoxia with a reduction at 48 to 72 h, whereas TNF-α increased progressively after 24 to 72 h. Acute exposure to hyperoxia (72 h) has been shown to induce lung inflammation and injury, impairing respiratory function, whereas prolonged exposure (96–120 h) causes lethality in rodents [35]. Hyperoxia exposure has been widely used as an experimental model for ALI or ARDS because of their similar pathologic features [35].

After 72 h of hyperoxia, lungs pretreated with either a low (12.5 μg/g) or high (100 μg/g) dose of aspirin for 3 days exhibited lower macrophages compared with those in the mock group. ROS are well known to be involved in physiological and pathophysiological processes. High levels of ROS are considered toxic, causing cell damage and cell death [36]. Hyperoxia produces copious ROS, and pretreatment with either a low (12.5 μg/g) or high (100 μg/g) dose of aspirin significantly reduces the numbers of macrophages and ROS production. Thus, pretreatment with aspirin could protect against hyperoxia-induced ALI and exert notable protective effects. In addition, pretreatment with a low or high dose of aspirin substantially reduced hyperoxia-induced macrophages and the protein expression levels of phosphorylated protein kinase B, NF-κB, interleukin 6 and TNF-α compared with hyperoxia treatment alone. These results indicate that aspirin pretreatment affects inflammatory response. Therefore, aspirin pretreatment significantly reduces neutrophil infiltration and lung edema compared with treatment with hyperoxia alone. Hamid et al. [37] also showed that healthy volunteers receive 75 or 1200 mg aspirin for seven days prior to lipopolysaccharide (LPS) inhalation, high-dose and low-dose aspirin both could decrease pulmonary neutrophilia, tissue damaging neutrophil proteases and BALF concentrations of TNF-α.

This suggests that pretreatment with aspirin could reduce NF-κB expression, demonstrating excellent protective effects against ALI and systemic oxidative stress caused by hyperoxia (FiO_2_ > 95%). Therefore, aspirin could protect lungs against hyperoxia-induced acute lung injuries in mice, and this protective effect may be correlated with anti-inflammatory and ROS-suppressive effects. In human research, due to the heterogeneity of the presentation, course and outcomes among patients meeting the clinical definition for ARDS, the results of the aspirin treatment are controversial. Hamid et al. [37] showed that aspirin inhibits pulmonary neutrophilic inflammation, at both low and high doses in bronchoalveolar lavage. Studies also showed that prehospital aspirin use was independently associated with a decreased risk of ARDS even after adjusting for the propensity of pre-hospital aspirin use [26] and was associated with a reduced risk of ICU mortality [38]. However, Kor et al. [39] found that the use of aspirin compared with placebo did not reduce the risk of development of ARDS at 7 days of hospitalization. As a result, further large-scale study may be required to identify which subgroup of ARDS may be beneficial from the aspirin treatment in the future.

This study had several strengths, including aspirin being easily available and cheap and the effects and mechanisms of aspirin in ARDS are clear in the animal model. However, our study also had some limitations, including only the animal study and the dose of aspirin is not clear to provide to humans. These limitations attenuate our ability to generalize conclusions about the effects of curcumin on ARDS.

## Abbreviations

ALI	Acute lung injury
ARDS	Acute respiratory distress syndrome
BALF	Bronchoalveolar lavage fluid
CC10	Clara cell 10 kilodalton protein
FVB	Friend leukemia virus B
H&E	Hematoxylin and eosin
IVIS	In vivo imaging system
LPS	Lipopolysaccharide
MMP	Matrix metalloproteinase
NRP-1	Neuropilin 1
PBS	Phosphate buffered saline
ROS	Reactive oxygen species
